# A Simple Retroelement Based Knock-Down System in *Dictyostelium*: Further Insights into RNA Interference Mechanisms

**DOI:** 10.1371/journal.pone.0131271

**Published:** 2015-06-25

**Authors:** Michael Friedrich, Doreen Meier, Isabelle Schuster, Wolfgang Nellen

**Affiliations:** Abt. Genetik, FB 10, Universität Kassel, Kassel, Germany; Max-Planck-Institute for Terrestrial Microbiology, GERMANY

## Abstract

**Characteristics of DIRS-1 Mediated Knock-Downs:**

We have previously shown that the most abundant *Dictyostelium discoideum* retroelement DIRS-1 is suppressed by RNAi mechanisms. Here we provide evidence that both inverted terminal repeats have strong promoter activity and that bidirectional expression apparently generates a substrate for Dicer. A cassette containing the inverted terminal repeats and a fragment of a gene of interest was sufficient to activate the RNAi response, resulting in the generation of ~21 nt siRNAs, a reduction of mRNA and protein expression of the respective endogene. Surprisingly, no transitivity was observed on the endogene. This was in contrast to previous observations, where endogenous siRNAs caused spreading on an artificial transgene. Knock-down was successful on seven target genes that we examined. In three cases a phenotypic analysis proved the efficiency of the approach. One of the target genes was apparently essential because no knock-out could be obtained; the RNAi mediated knock-down, however, resulted in a very slow growing culture indicating a still viable reduction of gene expression.

**Advantages of the DIRS-1–RNAi System:**

The knock-down system required a short DNA fragment (~400 bp) of the target gene as an initial trigger. Further siRNAs were generated by RdRPs since we have shown some siRNAs with a 5’-triphosphate group. Extrachromosomal vectors facilitate the procedure and allowed for molecular and phenotypic analysis within one week. The system provides an efficient and rapid method to reduce protein levels including those of essential genes.

## Introduction

The social amoeba *Dictyostelium discoideum* is a model organism to study diverse aspects of cell biology, developmental biology, signal transduction and gene expression. A major advantage is the relatively easy tractability of the organism for genetic manipulations.

DIRS–1 represents the most abundant retrotransposable element in the model organism *D*. *discoideum* [1]. It belongs to the group of tyrosine recombinase (YR)-encoding elements that use a YR instead of an integrase for integration into the host genome. This group is characterized by a unique structure composed of three open reading frames (ORFs) flanked by inverted terminal repeats (ITR). ORF1 encodes a putative GAG protein while the second ORF contains the YR. ORF3 is composed of a reverse transcriptase (RT), a RNase H (RH), and a methyltransferase domain (MT) [2].

The ITRs, are not absolutely identical and the right ITRs contain a 27 bp extension [3,4]. ITRs usually bear numerous functions for gene expression: enhancer motifs, promoter activity, transcription initiation sites, transcription terminator, and polyadenylation signal [5]. The 5`-ITR acts as an RNA Polymerase II promoter while the 3`-ITR harbours a transcription termination site and a polyadenylation signal (reviewed in [6]).

However, the ITRs of the DIRS–1 retroelement are believed to serve as promoters in both directions, the left ITR for sense transcription and the right ITR for full length antisense transcripts [7] as well as for the previously reported stress induced antisense transcript E1 [8]. Bidirectional transcription may lead to the formation of a duplex RNA which could be the origin of endogenous small interfering RNAs (siRNA) to silence the retrotransposon DIRS–1 on the posttranscriptional level. This is supported by the fact that most siRNAs in *D*. *discoideum* are derived from DIRS–1 [9] and that the loss of DIRS–1 siRNAs results in the accumulation of DIRS–1 transcripts and the expression of DIRS–1 encoded proteins [10].

We here investigated if bidirectional transcription from the ITRs was sufficient to mediate an RNAi response and could eventually be used for gene knock-downs.

It is relatively easy to generate knock-out mutants in *D*. *discoideum* by homologous recombination [11–13]. However, there are numerous essential genes like protein kinases (e.g. casK), cofilins (cofA) or WASP proteins (wasA) for which knock-outs could not be obtained because of their lethal phenotype [14–16]. In addition, the laboratory strains AX3 and AX4 carry a large duplication on chromosome 2 [17] and other duplications of 15 kb and more have been reported in the genome [18]. Disruptions of genes in these regions are therefore not easy to obtain. Furthermore, knock-out attempts have been reported to sometimes result in a knocked-out copy and a duplicated intact copy of the gene [19]. Therefore, gene deletions or disruptions are sometimes laborious.

Knock-down strategies that may circumvent these problems have previously been reported using antisense [20] or hairpin constructs [21–23]. Driving sense and antisense RNA from opposing promoters has also been employed with variable and mostly unsatisfactory results. For unknown reasons, knock-downs show different efficiency depending on the target and the strategy. Therefore multiple approaches may be required to obtain successful silencing. Since the retrotransposon DIRS–1 was so efficiently silenced *in vivo* by RNAi we developed an easy to use knock-down system based on the features of DIRS–1. This system was functional for all the genes we have tested.

## Material and Methods

### Oligonucleotides

DNA oligonucleotides (Invitrogen) used in this study are listed in S1 Table.

### Cloning of the ITR contructs

For generation of the extrachromosomal vectors pDM_lITR_GFP and pDM_rITR_GFP, left and right ITRs were amplified by PCR using the primers DM_132 and DM_133, DM_144 and DM_145, respectively. The actin15 promoter in pDM304 [24] was replaced by either the left or right ITR using the restriction sites XhoI/BglII. The GFP sequence was amplified using the primers DM_21 and DM_18. GFP was inserted using BglII/SpeI restriction sites.

For the extrachromosomal vector pDM_lITR_BglII/SpeI_rITR, the left ITR was amplified as described above. The right ITR was amplified by PCR using primers DM_134 and DM_135. The left ITR replaced the actin15 promoter of pDM304 using the restriction sites XhoI/BglII. The right ITR replaced the actin8 terminator using the restriction sites SpeI/HindIII.

For generation of the extrachromosomal pDM_act15_GFP_act15rv vector and pDM_act15_BglII/SpeI_act15rv, the actin15 promoter in reverse orientation (act15 rc) was amplified by the primers DM_204_SpeI act15 rc fw and DM_205_act15 rc HindIII rev. The actin8 terminator of pDM317 [24] was replaced by act15 rc using SpeI/HindIII to generate pDM_act15_GFP_act15rv. The actin8 terminator of pDM304 [24] was replaced by act15 rc using SpeI/HindIII to generate pDM_act15_BglII/SpeI_act15rv.

### Cloning of trigger sequences

From the selected genes (S2 Table), trigger sequences were amplified by PCR using primers as indicated in S1 Table. PCR products were cloned into vector pDM_lITR_BglII/SpeI_rITR or pDM_act15_BglII/SpeI_act15rv which both contained a unique BglII/SpeI restriction site in between the elements. Expression levels of the selected genes are given in S1 Fig.

### Growth and transformation of the amoeba

*Dictyostelium discoideum* Ax2 cells (strain 254) and derivatives were used for all experiments. Cells were grown axenically in HL5 (Formedia) containing 50 μg/mL ampicillin, 250 ng/mL amphotericin, 10 μg/mL penicillin and 10 μg/mL streptomycin.

Transformation with the extrachromosomal vectors [24] by electroporation was done as previously described [25]. Transformants were selected on media containing 10 μg G418/mL for 4 days. Subsequently, populations of transformants were analysed.

### Genes and strains

The Argonaute A knock-out strain was recently described [10] (accession number DDB_G0276299 (AgnA) www.dictybase.org [17]). Accession numbers of other genes used in this study are given in S2 Table.

For the analysis of knock-downs, two independently transformed populations were used.

### RNA isolation

Total RNA was isolated as described previously [7]. Total RNA was resuspended in an appropriate volume of dH_2_0 and the concentration was determined spectro-photomectrically by using a NanoDrop (Peqlab, Nanodrop 1000).

### Northern Blot analysis

Total RNA blotting. For detection of gene transcripts, 12 μg of total RNA were separated by electrophoresis in a 1,5% (w/v) agarose gel 1X MOPS (20 mM MOPS, 5 mM NaAc, 1mM EDTA, pH 7). Each RNA sample was denatured before loading in 4 μL 10X MOPS, 4μL 37% (w/v) formaldehyde and 20 μL formamide. Samples were heated to 65°C and mixed with 6X loading buffer before loading. After capillary transfer to a nylon membrane (Amersham Hybond-NX), UV cross-linking was carried out (312 nm, 0,560 J/cm^2^).

Small RNA blotting. For detection of small RNAs, 10 μg of total RNA were separated by electrophoresis in a 13% (w/v) polyacrylamide gel in 20 mM MOPS (pH 7) and 7M urea. After electroblotting onto a nylon membrane for 10 min at 20 V (Amersham Hybond-N), RNA was chemically cross-linked as described previously [26].

Hybridization**.** Pre-hybridization and hybridization were carried out in Church buffer [27]. Oligonucleotide probes were labelled by transfer of ^32^P from the γ position of ATP to the 5´-hydroxyl terminus of single-stranded DNA catalysed by the T4 Polynucleotide Kinase (Thermo Fisher Scientific). Probes are listed in S1 Table. All blots were washed as described previously [9]. Hybridization signals were detected after exposure to an image plate and read-out on a phosphor imager (FLA-7000, Fujifilm).

### Western Blot & quantification

5x10^6^ cells were pelleted, resuspended in 100 μL 1X Laemmli buffer [28] and boiled for 5 min. 3 μl of the soluble supernatant was separated by SDS-PAGE and transferred onto a nitrocellulose membrane by semidry blot. The membrane was blocked with 5% (w/v) milk powder in phosphate buffered saline. For detection of proteins, monoclonal antibodies against GFP mAb 264-449-2 (Millipore), Severin (sevA) mAb 42-65-11 [29] Coronin (corA) mAb 176-3-6 [30] and alpha-Actinin (abpA) mAb 47-19-2 [31] were used. As secondary antibody, a horseradish peroxidase conjugated goat α-mouse antibody (Dianova) was used. Chemiluminescent blots were imaged on film and then scanned. For densitometric analysis of western blot signal intensity ImageJ software [32] was used.

### Growth assay

*Dictyostelium* Ax2 wild type and transformed cell lines were grown to exponential phase in axenic liquid media, complemented with the appropriate antibiotics. Cells were diluted to a density of 5x10^4^ cells per mL in fresh medium. Cell numbers were determined using a Beckman Coulter Z2 Cell and Particle Counter at 12–hour intervals over a period of 72 hours.

### Fluorescence microscopy

Cells were grown in axenic media to 1–2x10^6^ cells per mL. 100–200 μl of culture were applied on a coverslip, after cells had settled they were washed twice with Soerensen’s phosphate buffer, pH 6 [33] and fixed with 4% (w/v) paraformaldehyde dissolved in Soerensen’s phosphate buffer for 10 min followed by cold methanol (-20°C) for 20 to 60 min. Afterwards coverslips were washed twice with PBS and nuclei were stained with DAPI. Finally cells were mounted on slides in one drop of gelvatol mounting medium.

Nuclei of 200 cells of two independent transformations of each strain were counted. All pictures were taken with a Leica DM IRB inverted fluorescence microscope (Leica, Wetzlar, Germany).

### Terminator 5′-Phosphate-dependent Exonuclease assay

Exonuclease digest was performed according to the manufacture’s protocol (Epicentre Technologies Corp., Chicago, USA). In brief, total RNA (10 μg) was incubated with reaction components (1U Terminator Exonuclease in buffer). For buffer A (maximum 5′-monophosphate RNA digestion), the reaction was incubated at 30°C for 1 h. Reaction with buffer B (minimum of non-5′-monophosphate-specific activity) was incubated at 42°C for 30 min. The reactions were terminated by adding 1 μl 100 mM EDTA (pH 8).

## Results

### 1. DIRS-1 ITRs function as strong promoters

The ITRs of the retroelement DIRS–1 are believed to serve as promoters, the left ITR for sense and the right ITR for antisense transcripts.

**Fig 1:**
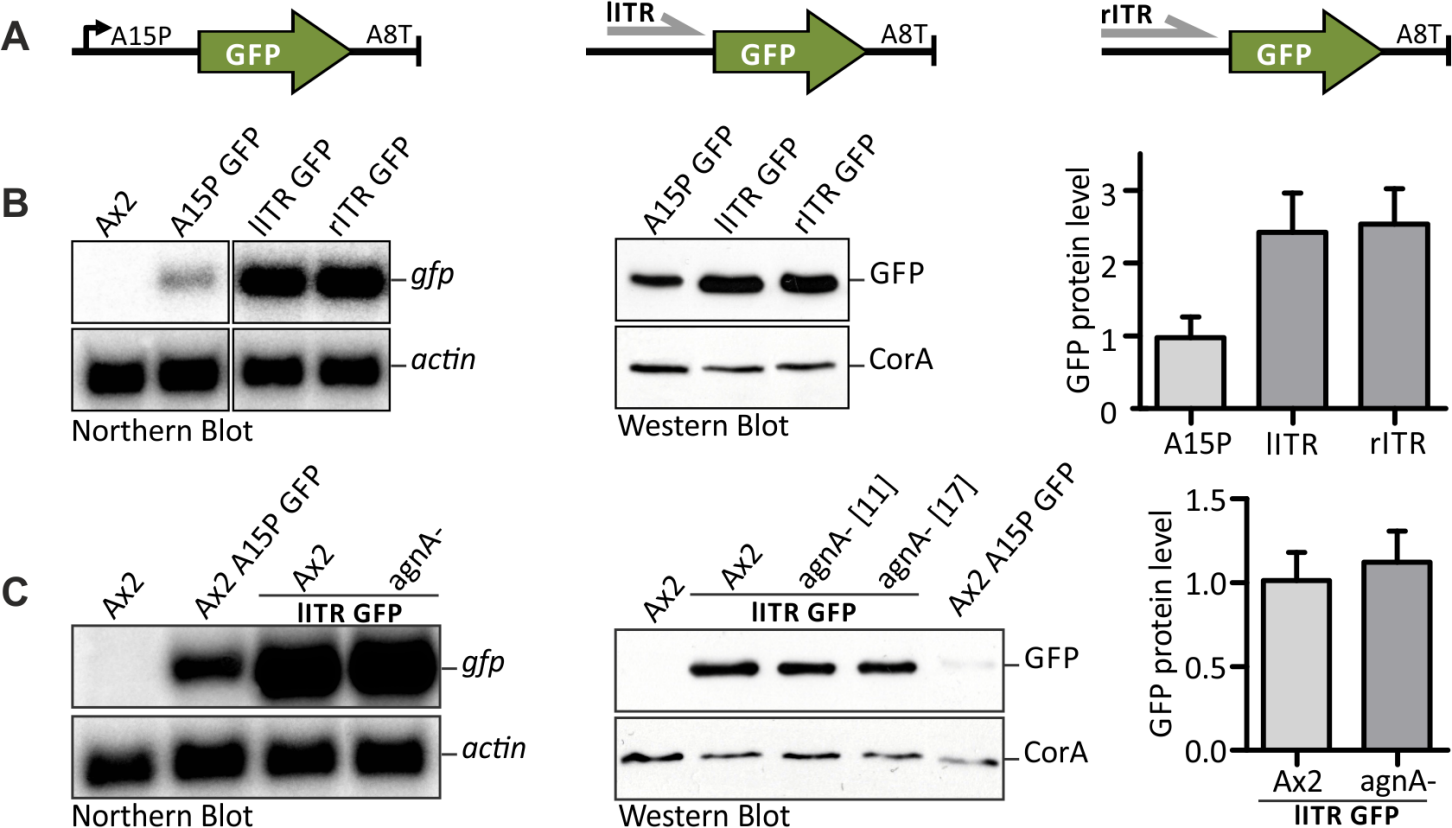
Analysis of ITR promoter activity. (A) The GFP coding region was inserted between actin15 promoter (A15P) and actin8 terminator (A8T), DIRS–1 left ITR (lITR) and A8T, inverted DIRS-1 right ITR (rITR) and A8T. (B) Detection of GFP mRNA expression in Ax2 wild type driven by the constructs depicted in Fig 1A. As a loading control, the blot was rehybridised with a ^32^P labelled oligonucleotide directed against actin (left panel). Western blot shows GFP protein expression in Ax2 wild type driven by the constructs depicted in Fig 1A. Coronin A (CorA) was used as a loading control (middle panel). The right panel shows the corresponding quantification of Western blots. Analysis was performed using ImageJ. Ax2 A15 GFP: n = 8. Ax2 lITR GFP: n = 4. Ax2 rITR GFP: n = 4. Error bars: mean with SD. (C) Detection of mRNA expression (left) and protein level (middle) for GFP driven by the lITR in Ax2 wild type and agnA knock-out (agnA-) cell lines. Numbers in square brackets indicate numbers of independent populations. The right panel shows the corresponding quantification of Western blots. Analysis was performed using ImageJ. Similar results were obtained with the right ITR (data not shown). Ax2 lITR GFP: n = 4. agnA- lITR GFP: n = 4. Error bars: mean with SD.

We tested the coding region of GFP with both ITRs as promoters and the actin8 terminator (Fig 1A). Northern blots revealed a much stronger accumulation of GFP mRNA driven by the left or right ITR relative to the frequently used actin15 promoter (Fig 1B). These findings were also confirmed on the protein level where both ITRs led to a more than 2–fold stronger expression than the actin15 promoter (Fig 1B).

We have previously described that the Argonaute A (AgnA) protein was required for regulation of DIRS–1 [10]. Accumulation of GFP from constructs containing the left or right ITR was independent of agnA indicating that the Argonaute had no significant effect on transcription (Fig 1C).

### 2. Opposing DIRS–1 ITRs lead to siRNAs and downregulation of genes

**Fig 2:**
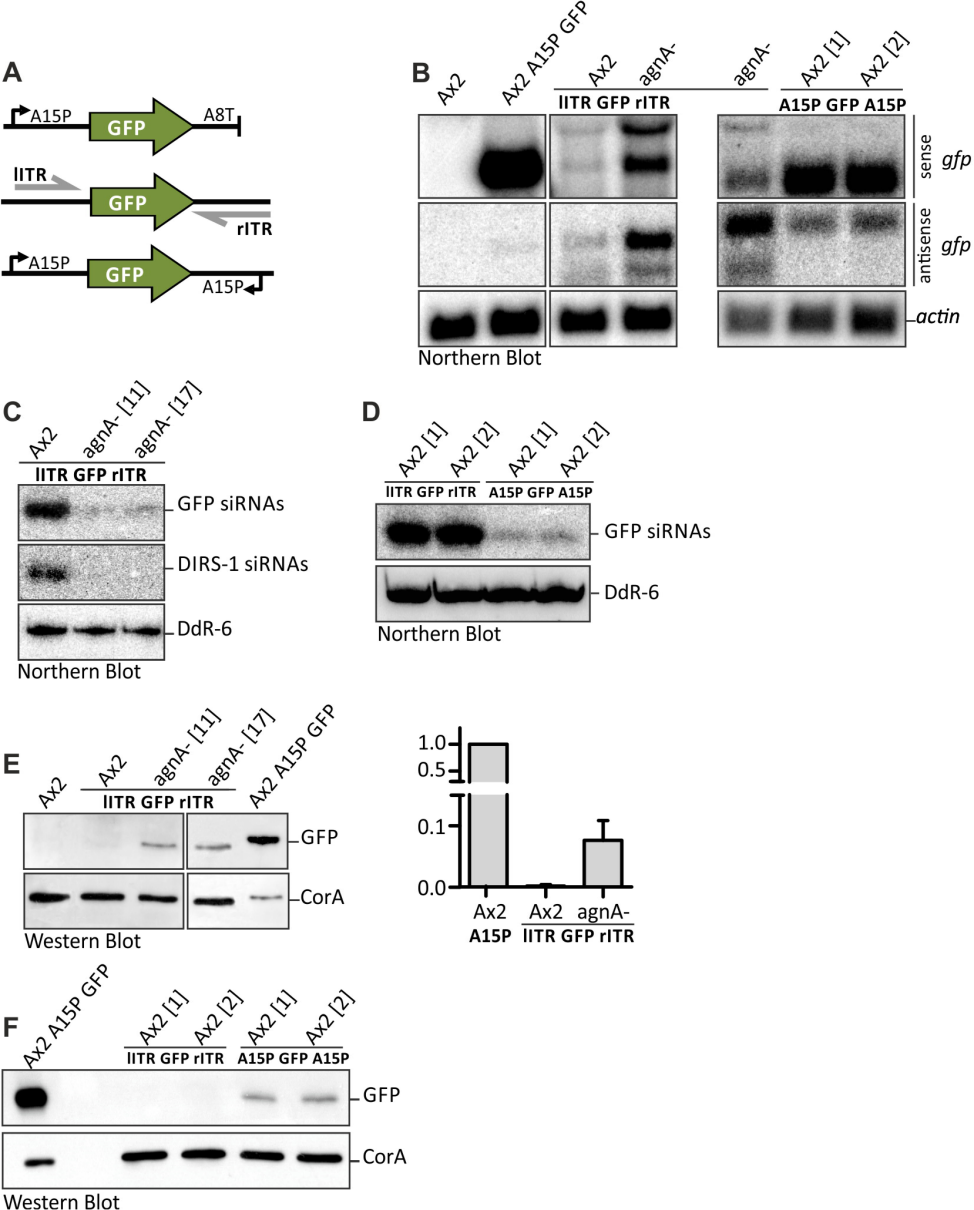
GFP RNA and protein levels from bidirectional promoter constructs. (A) Schematic presentation of the GFP reporter constructs. The GFP coding region was inserted between lITR and rITR of DIRS–1 and between opposing actin15 promoters (A15P). As a control, GFP with flanking A15P and A8 terminator (A8T) was used. (B) Analysis of GFP mRNA by Northern blots in the indicated strains. Blots were hybridized with probes against GFP sense mRNA (sense gfp, probe P29) and antisense mRNA (antisense gfp, probe P30). Equal loading was verified by rehybridization of the membrane with a ^32^P labelled oligonucleotide directed against actin. Samples for A15P GFP A15P were run on a different gel and hybridized separately (see actin loading control). (C) Representative Northern blot of GFP and DIRS–1 siRNAs in the indicated strains. Equal loading was verified by rehybridization of membrane with a ^32^P labelled probe directed against snoRNA DdR-6. (D) Representative Northern blot comparing GFP siRNAs generated from opposing ITRs and opposing A15 promoters. DdR-6 was used as a loading control. (E) Representative Western blot of GFP expression under the control of the left and right ITR in the indicated strains. GFP expression by A15P (¼ was loaded to avoid overexposure of the GFP signal) was used as a positive, the untransformed Ax2 as a negative control. CorA was used as a loading control. Quantification of the GFP intensity was performed using ImageJ. GFP expression by A15P was set to 1 (right panel). Ax2 lITR GFP rITR: n = 2. agnA- lITR GFP rITR: n = 4. Error bars: mean with SD. (F) Comparison of GFP silencing by opposing ITRs and A15Ps. GFP expression by A15P (¼ was loaded to avoid overexposure of the GFP signal) was used as a positive control.

Since DIRS–1 is controlled by siRNAs, which originate from the antiparallel transcripts, we assumed that any sequence cloned between the ITRs might produce siRNAs and silence expression of the corresponding gene *in cis* (Fig 2A). As shown in Fig 2 this was indeed the case: the mRNA levels were strongly reduced and GFP expression was barely detectable in wild type cells (Fig 2B and 2E). Simultaneously, we detected substantial amounts of siRNAs directed against GFP (Fig 2C).

We recently demonstrated that AgnA is essential for proper DIRS–1 siRNA generation and silencing of the retrotransposon [10]. As expected, only minor amounts of siRNAs were observed for GFP and DIRS–1 likewise in agnA- cells and gene silencing was reduced for the GFP reporter construct (Fig 2B, 2C and 2E). We detected two sense as well as two antisense gfp transcripts in agnA- cell lines and to a lower extent in the wild type cells. Relative to gfp mRNA terminated by the commonly used actin8 terminator these transcripts seemed to be larger, probably due to different polyadenylation sites within the ITRs (Fig 2B). Furthermore GFP was detectable by Western blot analysis in agnA- cells but not in the wild type background (Fig 2E).

It should be noted, that in the agnA- strain, RNAi mediated gene silencing was not completely abolished (see minor amounts of residual siRNAs in Fig 2C). Furthermore, opposing promoters may reduce transcript levels by transcriptional interference (see discussion). For comparison, we inserted the GFP coding region between two opposing actin15 promoters (Fig 2A). Even though silencing was observed, it was much weaker than with using the ITRs. Sense as well as antisense transcripts accumulate to relatively high level (Fig 2B) but there were only marginal amounts of siRNAs detectable (Fig 2D). Apparently, the complementary transcripts do not efficiently form a Dicer substrate for generating siRNAs thus resulting in the accumulation of GFP protein in wild type cells (Fig 2F).

Different combinations of ITRs and actin15 promoters to the left and the right of GFP showed various amounts of sense and antisense transcripts in agreement with the observation that the ITRs are stronger promoters than actin15. Silencing was always much less pronounced than with the combination of left and right ITR (data not shown).

### 3. Bidirectional transcription of trigger sequences knocks down gene expression *in trans*

Based on these observations, we speculated that siRNAs may also work *in trans* when endogene sequences were used as triggers in between the ITRs.

**Fig 3:**
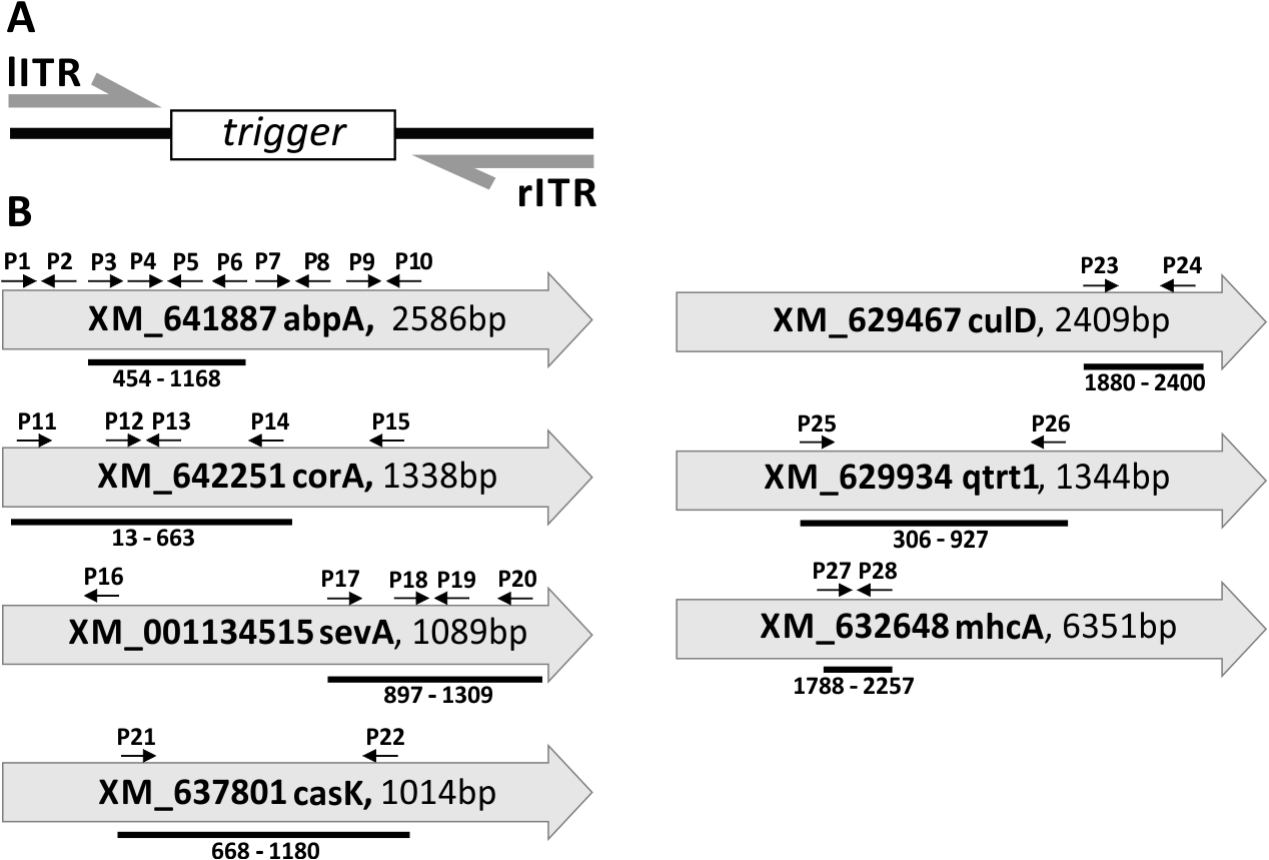
Schematic presentation of the knock-down system. (A) A gene fragment is inserted in between the left and right ITR as a trigger sequence. (B) Genes used for knock-down analysis are presented as mRNA with accession number and sequence length. Trigger sequences are indicated by black bars with position of the first and last nucleotide in respect to the ATG start. Oligonucleotides and their orientation used as hybridization probes are indicated by black arrows.

We have chosen the *D*. *discoideum* genes alpha actinin A (abpA), coronin A (corA), severin A (sevA), casein kinase (casK), cullin D (culD), queuine tRNA-ribosyl transferase 1 (qtrt1) and myosin heavy chain A (mhcA) for proof of principle experiments. Using the extrachromosomal vector pDM_lITR_BglII/SpeI_rITR, we cloned fragments (triggers) of 400 to 700 bp into the BglII/SpeI site. Fig 3 shows the structure of the mRNA, the size and position of the cloned fragments as well as the probes used in this study. None of the fragments had an ATG start codon in a reasonable reading frame.

**Fig 4:**
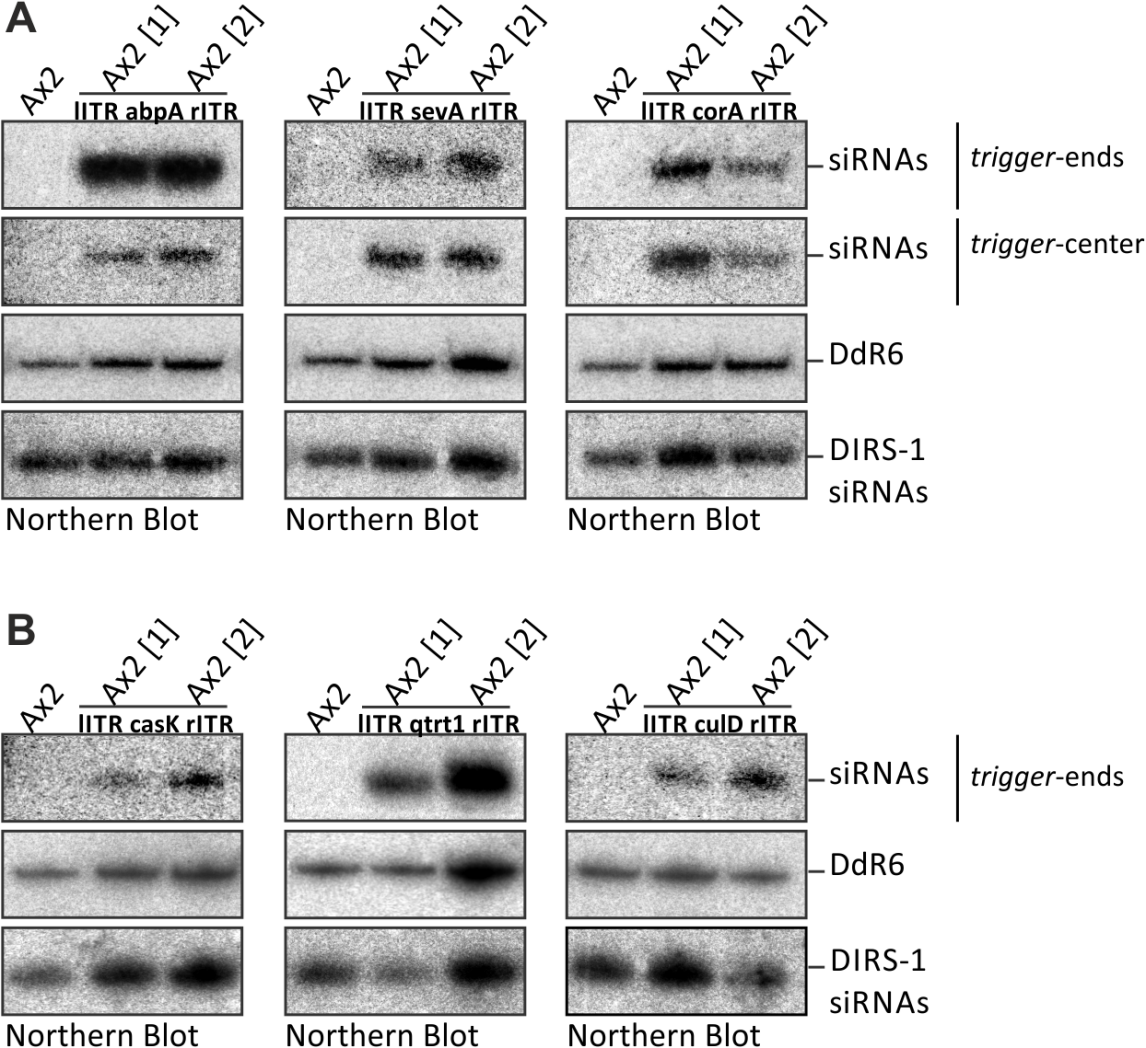
Detection of siRNAs based on the bidirectional RNA expression driven by the left and right DIRS-1 ITRs. (A) Northern blot to detect alpha-Actinin (abpA), Coronin (corA) and Severin (sevA) siRNAs. Probes at the trigger-ends and near the trigger-centres are indicated and correspond to the probes shown in Fig 3 (see also S1 Table). (B) Northern blot to detect casein kinase (casK), cullin D (culD), queuine tRNA-ribosyl transferase 1 (qtrt1) siRNAs at the end of the trigger. A mixture of radiolabelled oligonucleotides (position indicated in Fig 3, see also S1 Table) was used for hybridization. All samples in A and B respectively were run on the same gel. Probes against snoRNA DdR-6 and DIRS–1 siRNAs were separately used as loading controls for A and B.

Northern blots revealed that siRNAs, which were not detectable in the wild type, were generated from all constructs (Fig 4, data not shown for mhcA). For unknown reasons, the amounts of siRNAs were very different but did not vary much between biological replicates. The amount of siRNAs seemed to be independent of the target expression level (S1 Fig) and did not correlate with length or position of the trigger. Hybridisation was done with different oligonucleotides as probes (S1 Table). This revealed that siRNAs are present at the end as well as near the centre of the generated trigger fragments with some changes in their relative amounts (Fig 4A).

### 4. siRNAs show reduced or no transitivity *in trans*

To examine if the trigger derived siRNAs could mediate spreading, i.e. RNA dependent RNA polymerase (RdRP) activity, on the endogenous target gene, we examined siRNAs on sequences flanking the trigger region. These would be derived from transitivity along the target gene in 5’ or 3’ direction as we have previously shown [10].

While siRNAs were readily detected by probes to the very end of the trigger (Fig 4A and 4B), no secondary siRNAs were observed either in 3’ or in 5’ direction (data not shown). This indicated that siRNAs derived from the transgene (trigger) were not able to mediate spreading into adjacent sequences on the target endogene or were at least very inefficient to do so.

### 5. Secondary siRNAs are generated on targets *in cis* as well as *in trans*

**Fig 5:**
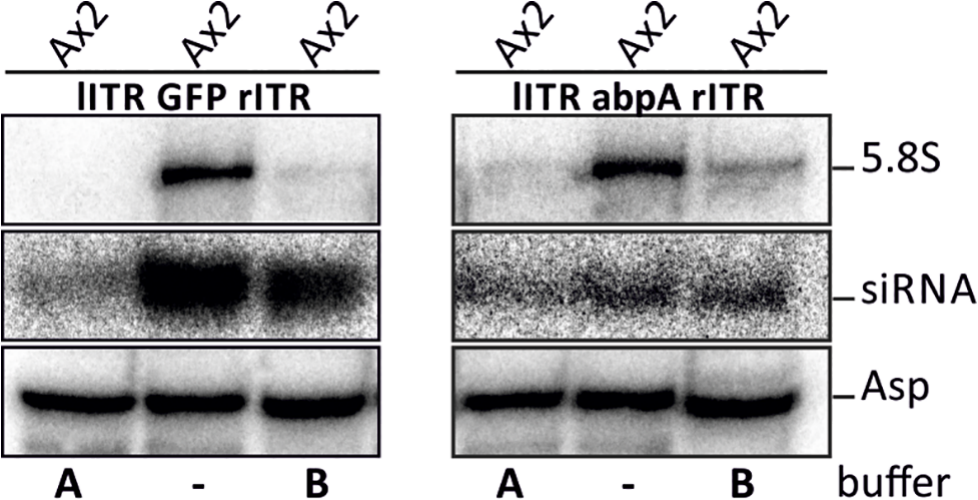
Determination of 5’ endgroups by Terminator 5'-phosphate-dependent exonuclease (5'exo). RNA samples of the ITR GFP strains (silencing *in cis*) and ITR abpA strains (silencing *in trans*) were digested with 5'exo, in buffer A and buffer B as indicated, separated on an acrylamide gel, blotted and probed with radiolabelled oligonucleotides complementary to 5.8S (digestion control), tRNA^Asp^ (control for undigested RNA) and for GFP and abpA siRNAs respectively.

To examine the nature of siRNAs, we estimated the amount of RNAs with a 5’-monophosphate and a 5’-triphosphate. A 5’-triphosphate can only be generated at the beginning of an RdRP product. Terminator 5'-phosphate-dependent exonuclease (5'exo) is a 5’ to 3’ exonuclease which is essentially blocked by a triphosphate end. We have used two assay conditions suggested by the manufacturer: buffer A which represents the standard protocol and buffer B which is milder and avoids rare degradation of RNAs containing a 5’-triphosphate. Fig 5 shows that the ribosomal 5.8S RNA was completely degraded in buffer A while residual amounts were left with buffer B. siRNAs of the *cis* target (GFP) were strongly reduced but not completely lost with buffer A suggesting that the remaining siRNAs were secondary and contain a 5’-triphosphate. For the *trans* target (abpA) it appears that the relative amount of secondary siRNAs was even higher. tRNAs are not digested by 5'exo and probing for tRNA^Asp^ served as a control. We conclude that secondary siRNAs are generated in both cases but they do not display transitivity on the endogene *in trans*. Due to the different activities in buffers A and B we cannot reliably determine the relative amounts of siRNAs with a 5’-triphosphate but can only state their existence.

### 6. Trigger derived siRNAs silence expression of the corresponding endogenes

**Fig 6:**
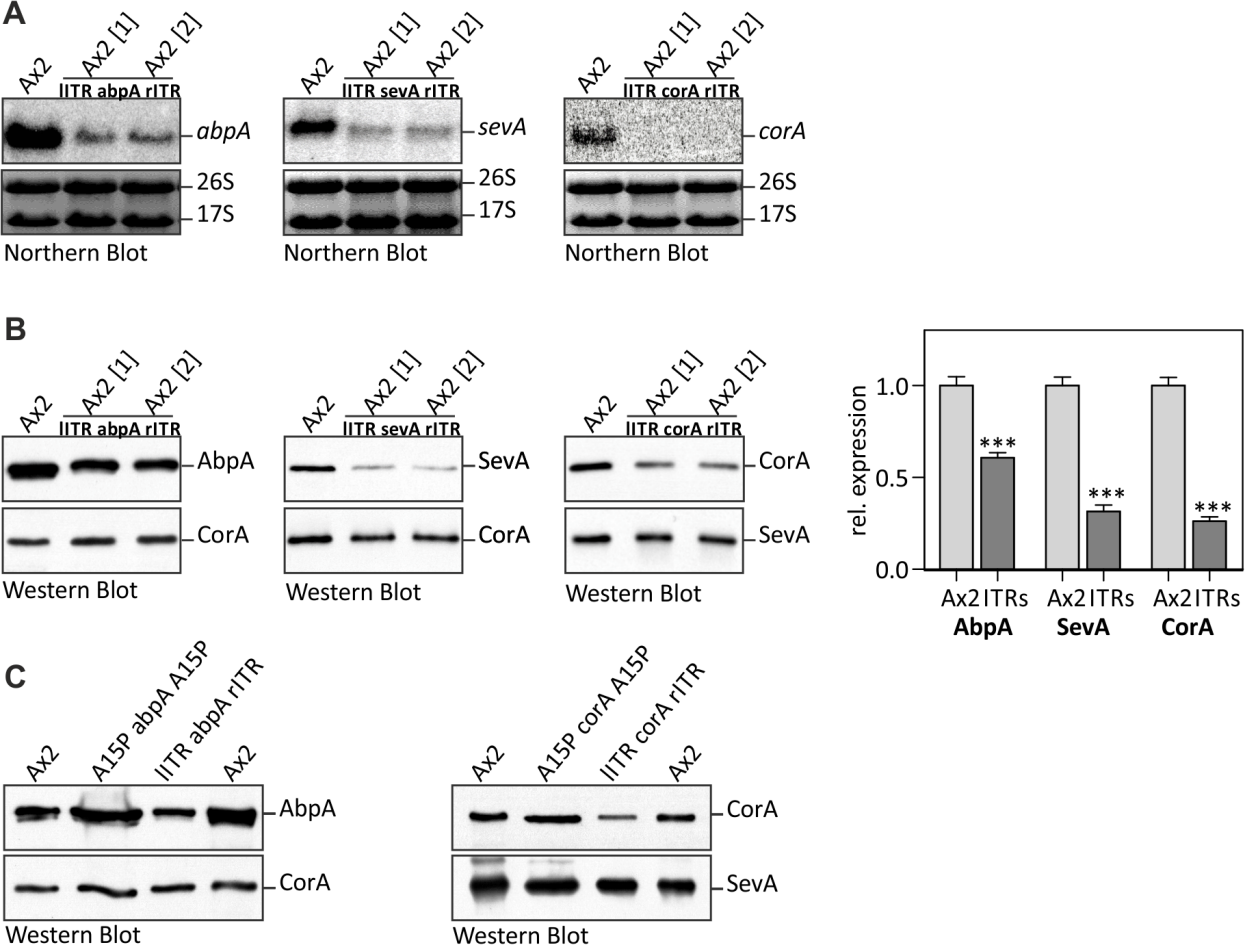
Reduced transcript and protein levels of knock-down targets. (A) Northern blot analysis for abpA, corA and sevA mRNA levels in the respective knock-down strains relative to the Ax2 wild type control. A mixture of radiolabelled probes outside the trigger region (P10, P15, P16; see Fig 3) was used for hybridization. Equal loading was verified by rRNA staining with ethidium bromide. All samples were run on the same gel. (B) Representative Western blot of AbpA, SevA and CorA protein expression in the Ax2 wild type and the relevant knock-down line (n = 2). CorA or SevA was used as loading control. Quantification of signal intensity relative to the respective control protein was performed using ImageJ. Ax2: n = 8. Ax2 ITRs: n = 8. Error bars: mean with SD. ***, p<0,001. (C) Comparison of *trans* silencing on the protein level with opposing A15Ps and ITRs for abpA and corA relative to Ax2 wild type. CorA or SevA were used as loading controls.

We then asked the question if the trigger derived siRNAs could silence the corresponding endogenous gene. We examined mRNA from abpA, sevA and corA since they were expressed at sufficiently high levels to be detected by Northern blots. The analysis revealed a strong reduction of transcript levels for these knock-down strains relative to the Ax2 wild type (Fig 6A) and simultaneously a reduction on the protein level (Fig 6B). Though we did not attempt to quantify the amounts of siRNAs, mRNAs and proteins, it was rather obvious that there was no strict correlation. All endogenes were partially silenced but with different efficiency.

For comparison, silencing was also assayed using bidirectional actin15 promoters which had shown reduced silencing *in cis*. Fig 6C shows that no significant *trans* silencing effect was observed with constructs directed against the corA and abpA genes.

### 7. Knock-downs by RNAi cause the expected phenotypes

**Fig 7:**
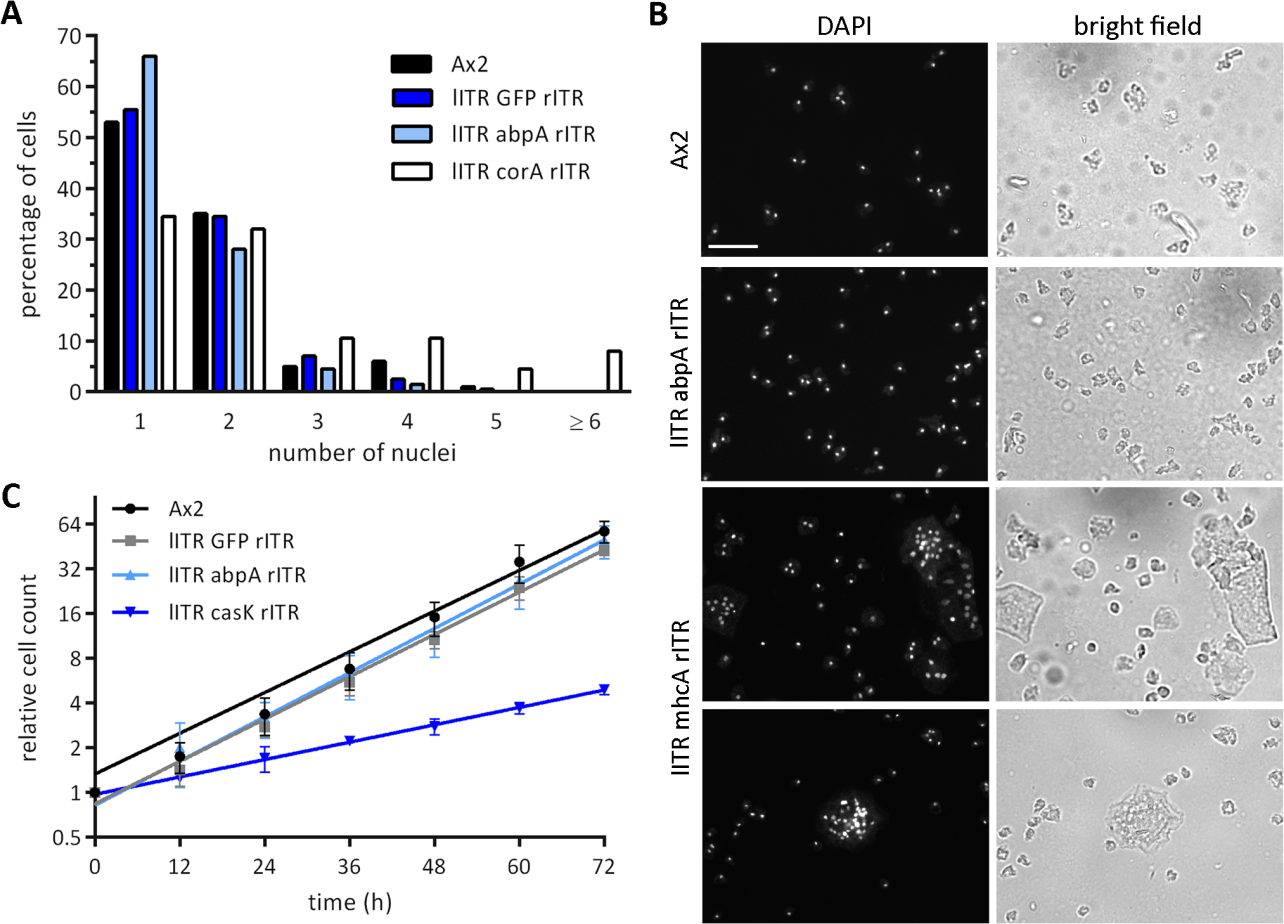
Comparison of published knock-out phenotypes with knock-downs by ITR constructs. (A) Nuclei count in Ax2 wild type cells and different knock-down strains. Knock-down of corA (lTR corA rITR) resulted in increased multinuclearity. 200 cells of each strain of two independent transformations were counted. (B) Knock-down of mhcA by an ITR construct showed huge cells containing up to 30 nuclei and more compared to Ax2 wild type and to a control knock-down of abpA. Scale bar 50μm. (C) Growth curve of Ax2 wild type cells and different knock-down strains. Knock-down of casK resulted in a slow growing culture. Measurement of cell density in the indicated cell lines over 72 hours. Data are plotted as relative cell count over time. Ax2, lITR GFP rITR, lITR abpA rITR: n = 2; lITR casK rITR: n = 4.

It was now of interest to see if the reduction of protein levels was sufficient to display knock-down phenotypes. For corA knock-out strains, multinuclearity during growth has been reported [34]. The corA knock-down strains showed a very similar phenotype in that the number of mononucleate cells was reduced and the number of multinucleate cells increased (Fig 7A, S2 Fig). Similarly, mhcA knock-down and knock-out cells are very large and have many nuclei [35,36]. The same phenotype as previously described was found upon expression of a mhcA ITR construct (Fig 7B).

Disruption of the casK gene is apparently lethal [15]. This was supported by our own unsuccessful attempts to knock the gene out by homologous recombination (data not shown). However, a casK knock-down strain could be obtained. Growth analysis showed that the cells had a significantly reduced doubling time, which suggested that the knock-down was successful and conferred a growth disadvantage (Fig 7C).

## Discussion

Though it was expected that the DIRS–1 ITRs both have promoter activity, we now provide experimental evidence that these relatively short sequence elements promote transcription significantly stronger than the commonly used actin15 promoter. DIRS–1 ITRs may thus be used as an alternative, especially when strong expression is desired.

Based on these strong opposing promoters of a genetic element that is naturally silenced by RNAi, we have established a convenient gene silencing system in extrachromosomal vectors which is easier to use and apparently more reliable than the previously described antisense and hairpin constructs [21,23]. We have tested seven genes and find RNAi effects on all of them: the generation of siRNAs, reduction on the mRNA and the protein level and expected phenotypes and combinations of all of the above wherever it was examined. Reasons for the good functionality may be the almost equal strength of the two ITR promoter activities but probably some additional intrinsic features of the ITR sequences that may attract the RNAi machinery. For example, minor amounts of endogenous siRNAs complementary to the ITRs are found *in vivo* under special conditions [10]. Since the trigger transcripts most likely extend into the ITRs they may be targets for this special minor class of endogenous siRNAs and possibly facilitate assembly of the Dicer complex. This may explain why similar trigger constructs with opposing actin15 promoters or actin15 and V18 promoters [23] did not result in significant endogene silencing.

In general, the generation of siRNAs caused a reduction in mRNA and protein levels. However, the amounts of siRNAs, mRNA and protein expression levels were not clearly correlated. We have previously shown that the amount of siRNAs (probably secondary siRNAs) depends on the presence and most likely the abundance of target transcripts [23]. Here, expression levels of the target genes (S1 Fig) as derived from DictyExpress [37,38], however, did not correlate well with the abundance of siRNAs. We rather assume that structural features of trigger transcripts facilitate or inhibit dsRNA formation [39] and that secondary structures of the target transcripts influence the accessibility for siRNAs.

For the detection of siRNAs we have used oligonucleotides very close to the ends of the trigger sequence and more towards the center. Hybridization signals were different, reflecting the unequal distribution of siRNAs along a target RNA [10,21]. This was most obvious for abpA siRNAs (Fig 4A). Nevertheless, the relative amounts of siRNAs for a given target were similar when compared with other targets, indicating that intrinsic features make a good or a bad RNAi target.

Interestingly, the reduction of mRNA abundance was usually more pronounced than the reduction on the protein level. This makes it unlikely that siRNAs have an additional effect on translation. It rather seems that the loss of mRNA maybe partially compensated by an enhancement of translation. Our findings are in contrast to experiments by Wiegand et al. with hairpin constructs against a transgene (ß–galactosidase) where only translational repression was observed [21]. They correspond, however, to our previous data with a hairpin construct against discoidin and a hairpin construct against ß–galactosidase where silencing occurred on the level of mRNA stability [23]. It should be noted that the construct against ß–galactosidase that was used by Wiegand and co-workers, differed in length and position from the one used in our previous study.

We have previously shown that AgnA is essential for the generation of (secondary) siRNAs and that RNAi mediated gene silencing is largely abolished when agnA is disrupted [10]. It was therefore surprising that expression of a GFP gene flanked by the ITRs was relatively low in the agnA- strain when compared to GFP driven by the actin15 promoter. As expected, GFP siRNAs were barely detectable in this strain. Though transcripts in both orientations were detected, antisense was apparently underrepresented. The formation of dsRNA seemed to be less efficient and one may assume that the primary Dicer products were rather unstable. In addition, other mechanisms may reduce the expression levels and thus the generation of siRNAs. These could be transcriptional interference (reviewed in [40]) or some other interaction between the opposing promoters.

**Fig 8:**
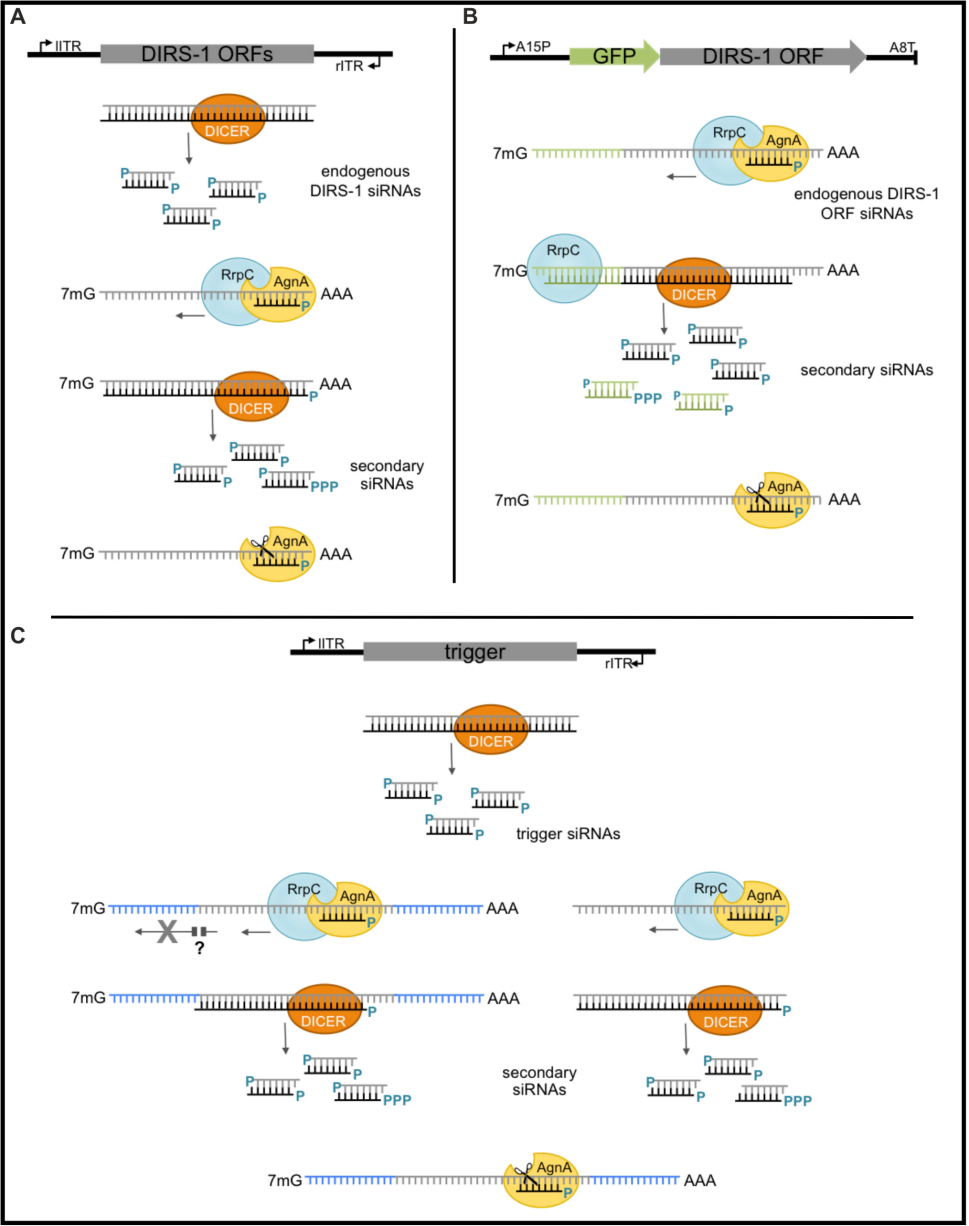
Model for gene silencing by natural and transgene derived siRNAs. (A) The endogenous DIRS–1 sequences are transcribed in both directions and generate dsRNAs (grey) which are cleaved by Dicer to ~21 nt siRNAs. siRNAs recruit RrpC in a AgnA-dependent manner. The resulting dsRNA which may be synthesised primer dependent or primer independent in 5’ and 3’ direction is processed to secondary siRNAs. Primer independent siRNAs carry a 5’-triphosphate. Argonaute proteins (probably AgnA) silence DIRS–1 by mRNA destabilisation [10]. (B) A transgene fusion of GFP and a DIRS-1 ORF is targeted by endogenous DIRS-1 siRNAs *in trans*. These may be primary or secondary siRNAs. They recruit the RdRP RrpC, which produces primer dependent and primer independent secondary siRNAs in 5’ and 3’ direction. Consequently, siRNAs matching adjacent sequences (here GFP) are generated. Silencing occurs on the level of mRNA destabilisation. DIRS–1 sequences are colour coded grey, GFP sequences green. Secondary siRNAs have a 5’-triphosphate or a monophosphate when they are Dicer products from longer dsRNAs or primer dependent. (C) siRNAs are generated from a bidirectionally transcribed trigger transgene. They can target the trigger transcripts *in cis* and complementary sequences on mRNAs of the corresponding endogene. They produce secondary siRNAs either from the trigger only or from both the target and the trigger transcript. If transgene derived siRNAs can only recruit RdRPs *in cis*, i.e. on the transgene itself, this could explain the lack of spreading along the mRNA of the endogene. Trigger sequences are colour coded grey, flanking sequences of the corresponding endogene blue.

Amplification as well as spreading of siRNAs along a target and beyond the trigger is mediated by RNA dependent RNA polymerases and we have previously shown that in *D*. *discoideum* RrpC is required for the generation of the majority of siRNAs. These secondary siRNAs start out with a 5’-triphosphate when the RdRP works independent of a siRNA primer. Depending on the processivity of the RdRP (which is not known in *D*. *discoideum*), multiple siRNAs can be diced from one RdRP product but only the first of these secondary siRNAs has a 5’-triphosphate. Secondary siRNAs were also generated from the ITR GFP constructs demonstrating that they not necessarily mediate spreading. Based on a rough estimate comparing total siRNAs, primary siRNAs (as they are found in agnA- strains) and siRNAs with a 5’-triphosphate, one may assume that the RdRP has a higher processivity than observed in *C*. *elegans* [41] and more secondary siRNAs are Dicer products (Fig 8B). Fire’s group has found that essentially all transitive siRNAs carry a 5’-triphosphate and are thus single 21mers produced by RdRPs. Due to experimental limitations of our assay system, an exact quantification of siRNAs with 5’-triphosphate is, however, not reliable.

In a different system using endogenous siRNAs to target tagged genes, spreading was observed at least up to 300 nts in 5’ and 3’ direction [10]. With transgene derived siRNA we did not see any spreading *in trans* at all (Fig 8C).

One may assume that secondary siRNAs are only generated on the *cis* sequence, i.e. on the sequence they are derived from. On the other hand, secondary, transitive siRNAs are readily found on fusion constructs, which are silenced by endogenous siRNAs derived from a different locus. Thus, transgene derived siRNAs were less efficient for transitivity on an endogene than endogenous siRNAs on a transgene [10]. We have so far no explanation for the different effects of siRNAs from different origins and no indication for differential modification that may distinguish between the species. In both cases we detect 5’-monophosphates and triphosphates and preliminary data (Boesler, unpublished) indicate that there is no 2’-O-methylation at all in *D*. *discoideum* siRNAs. This unexpected difference is depicted in the model in Fig 8.

Gene knock-downs by antisense (e.g. [20,36,42]) and by hairpin constructs (e.g. [21–23,43,44]) have previously been described. The system presented here may have several advantages. The construction of knock-down vectors is easier than that of hairpin constructs and involves only one PCR and one cloning step. Transformation with extrachromosomal vectors is rapid. At least with integrating plasmids we have previously seen various, mostly lower efficiencies and could e.g. not obtain a corA knock-down with an antisense or a hairpin construct in wild type cells [43].

We have not subcloned the transformants and may therefore underestimate the knock-down effect that can be obtained in single clones.

Using cDNA or genomic libraries in the ITR vector may provide an efficient system to screen for knock-down mutants.

Since DIRS–1 like elements are wide spread in various organisms, it will be of interest to see if the DIRS–1 derived knock-down system may also be applicable in these.

## Supporting Information

S1 Fig Expression levels of target genes. mRNA levels are based on RNA-seq from www.dictyexpress.org [37,38]. (DOCX)

S2 Fig Nuclei in corA knock-down cells. In corA knock-down strains, the number of multinucleate cells is increased compared to Ax2 wild type and control knock-down strains for abpA and GFP. Scale bar 10 μm. (DOCX)

S1 Table Oligonucleotides. (DOCX)

S2 Table Trigger sequences and gene names. (DOCX)
