# Supplementary material for: A Simple Retroelement Based Knock-Down System in Dictyostelium: Further Insights into RNA Interference Mechanisms
Source: PLoS One. 2015 Jun 25;10(6):e0131271. doi: 10.1371/journal.pone.0131271 (PMC4482531; doi:10.1371/journal.pone.0131271)
Supplement: S1 Fig — (DOCX) [file pone.0131271.s001.docx]

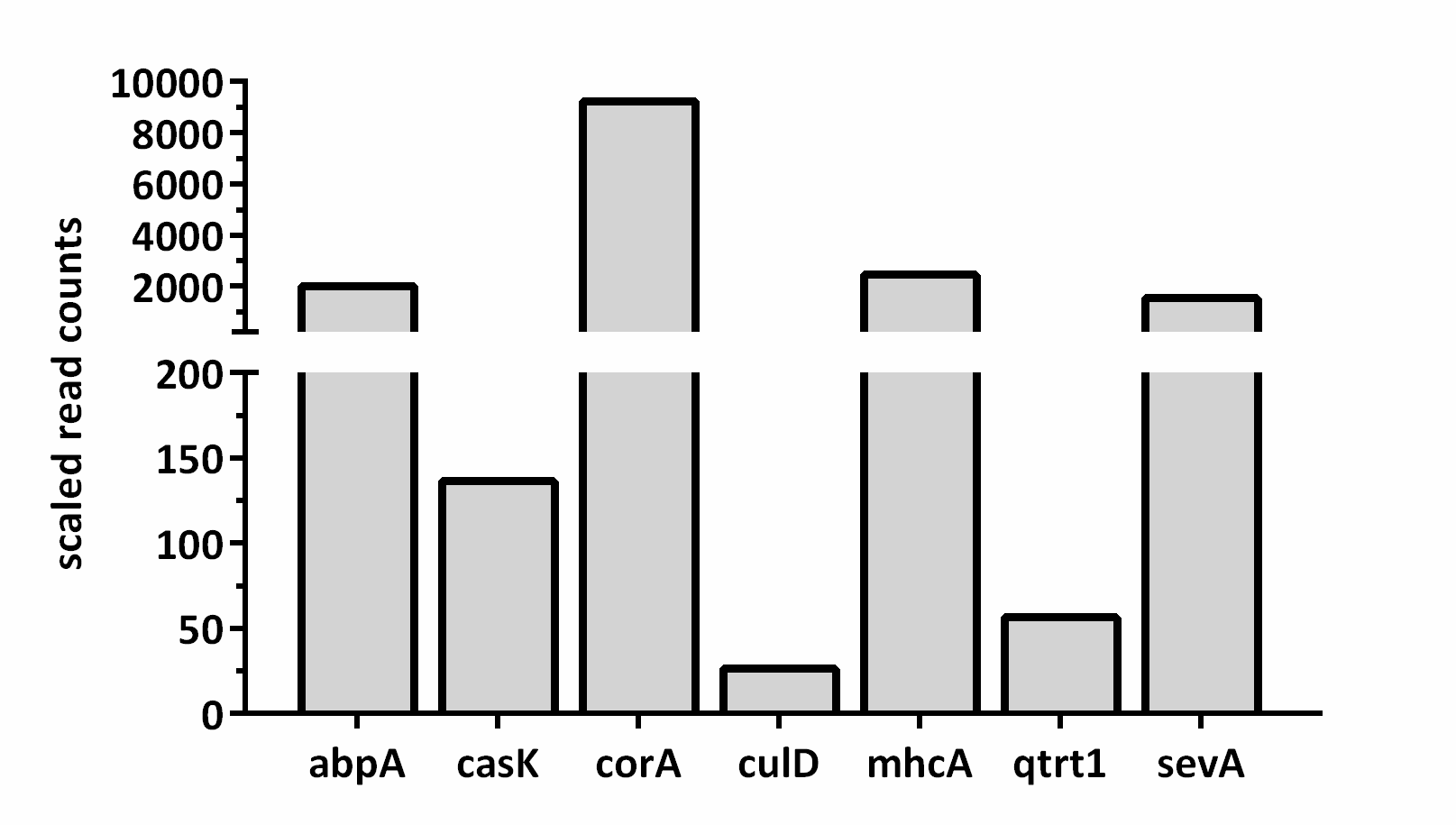


**Fig. S1:** *Expression levels of target genes.*

mRNA levels are based on RNA-seq from www.dictyexpress.org [[27](#_ENREF_27),[28](#_ENREF_28)].
