# Supplementary material for: A Simple Retroelement Based Knock-Down System in Dictyostelium: Further Insights into RNA Interference Mechanisms
Source: PLoS One. 2015 Jun 25;10(6):e0131271. doi: 10.1371/journal.pone.0131271 (PMC4482531; doi:10.1371/journal.pone.0131271)
Supplement: S2 Fig — (DOCX) [file pone.0131271.s002.docx]

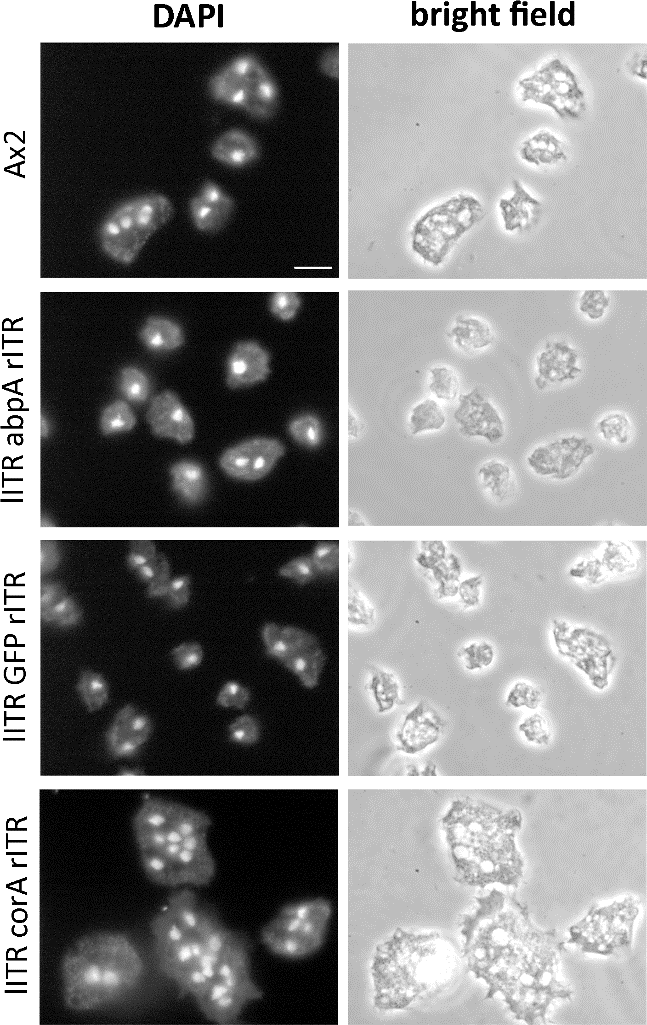


**Fig. S2: *Nuclei in corA knock-down cells.***

In corA knock-down strains, the number of multinucleate cells is increased compared to Ax2 wild type and control knock-down strains for abpA and GFP. Scale bar 10 µm.
