# Supplementary material for: A Simple Retroelement Based Knock-Down System in Dictyostelium: Further Insights into RNA Interference Mechanisms
Source: PLoS One. 2015 Jun 25;10(6):e0131271. doi: 10.1371/journal.pone.0131271 (PMC4482531; doi:10.1371/journal.pone.0131271)
Supplement: S1 Table — (DOCX) [file pone.0131271.s003.docx]

**Table S1:** Oligonucleotides

| **Primer name** | **Sequence** | **Description** |
| --- | --- | --- |
| MF_abpA_Fw_5spr. | CGTTTTGCCATTCAAGATATTTCA | probe **[P1]** |
| MF_abpA_Rv_5spr. | GGGCTTCTTTGGCACTCAATTCTTC | probe **[P2]** |
| MF_AbpA_Fw | AGATCTGATGACTTTGGGTATGATTTGG | Cloning trigger fragment,  probe (trigger-ends) **[P3]** |
| DM_182_abpA siRNAs fw | TTGGACAATCATTCTTCGTTTTGC | probe (trigger center) **[P4]** |
| DM_183_abpA siRNAs rev | AGCCAATTGTAAGTTACCAGCTTT | probe (trigger center) **[P5]** |
| MF_AbpA_Rv | ACTAGTGAGACCAGCTGGTGCAAC | Cloning trigger fragment,  probe (trigger-ends) **[P6]** |
| MF_abpA_Fw_3spr. | CAATCGTATTCTCAAGAAACTCG | probe **[P7]** |
| MF_abpA_Rv_3spr. | CAGATTTGGTGGTGGCCCAG | probe **[P8]** |
| DM_180_abpA qPCR primer fw | TTGGATTGAAGCTGCCGATG | probe **[P9]** |
| DM_181_abpA qPCR primer rev | GTGGAGGAAAGCGTCGAATT | probe **[P10]** |
| MF_CorA_Fw | AGATCTGTCCGTAGTAGTAAATATCGTCATG | Cloning trigger fragment,  probe (trigger-ends) **[P11]** |
| Coronin_qPCR_for | AAATATCGTCATGTTTTTGCAGCACAAC | probe (trigger center) **[P12]** |
| Coronin_qPCR_rev | AATAATGGAACTGATGTGGTTTTACCTGAA | probe (trigger center) **[P13]** |
| MF_CorA_Rv | ACTAGTGGCTCTGCTGTTTTTGACACC | Cloning trigger fragment,  probe (trigger-ends) **[P14]** |
| DM_179_corA qPCR primer rev | TTTGTGGGGTGGCAGATTTG | probe **[P15]** |
| DM_175_sevA qPCR primer rev | CAGTGCCAGCTTCATCTTGA | probe **[16]** |
| MF_SevA_Fw | AGATCTCCGCTGAAGTTGCACGTG | Cloning trigger fragment, probe (trigger-ends) **[P17]** |
| DM_184_sevA siRNAs fw | TTTTAGGTGGAAAAGGTGCAATCG | probe (trigger center) **[P18]** |
| DM_185_sevA siRNAs rev | TGATGATTTTGAACCGATCCAAGT | probe (trigger center) **[P19]** |
| MF_SevA_Rv | ACTAGTACCATTTTCTAAAACTCTAACTATTGG | Cloning trigger fragment, probe (trigger-ends) **[P20]** |
| IS_BglII_casK_for | agatctgaaggtgccaacattaaaaacaatg | Cloning trigger fragment, (trigger-ends) probe **[P21]** |
| IS_SpeI_casK_rev | actagtcataccagcaaacatacaaccaag | Cloning trigger fragment, (trigger-ends) probe **[P22]** |
| MF_CulD_Fw | AGATCTCTTTGAAATGGCAAAATGCTCTC | Cloning trigger fragment, (trigger-ends) probe **[P23]** |
| MF_CulD_Rv | ACTAGTGGGCTAAAGTTTTTCTGGTTTTC | Cloning trigger fragment, (trigger-ends) probe **[P24]** |
| IS_BglII_qtrt1_for | agatctgggattgacatcacagcaattgg | Cloning trigger fragment, (trigger-ends) probe **[P25]** |
| IS_SpeI_qtrt1_rev | actagtgccgacacccatgagataacg | Cloning trigger fragment, (trigger-ends) probe **[P26]** |
| IS_BglII_mhcA_for | AGATCTCGAACTTTGCTTCAAAG | Cloning trigger fragment, (trigger-ends) probe **[P27]** |
| IS_SpeI_mhcA_rev | ACTAGTcacgagcttcttcaatacgagc | Cloning trigger fragment, (trigger-ends) probe **[P28]** |
| DM_18_GFP_Rv | ACTAGTTTACTTGTATAGTTCATCCATGCCATGTG | Amplification of GFP, probe **[P29]** |
| DM_21_GFP_Fw | AGATCTATGGGTAAAGGAGAAGAACTTTTCACTGG | Amplification of GFP, probe **[P30]** |
| DM_132_XhoI DIRS-1 lLTR fw | CTCGAGTTTATATTATCATATATATATATATATTATG | Cloning left LTR DIRS-1 |
| DM_133_DIRS-1 lLTR BglII rev | AGATCTGAATTCCCAAATAAAAATGGTTTTAG | Cloning left LTR DIRS-1 |
| DM_134_SpeI_DIRS-1 rLTR fw | ACTAGTGAATTCATAAATCAAATTGTTTTAG | Cloning right LTR DIRS-1 |
| DM_135_DIRS-1 rLTR HindIII rev | AAGCTTTTAAAAATTTAATTTATTAAATTATATTTTA | Cloning right LTR DIRS-1 |
| DM_144_XhoI right LTR_fw | CTCGAGTTAAAAATTTAATTTATTAAATTATATTTTA | Cloning right LTR DIRS-1 (reverse, complement) |
| DM_145_right LTR rc BglII_rev | AGATCTGAATTCATAAATCAAATTGTTTTAG | Cloning right LTR DIRS-1 (reverse, complement) |
| DM_204_SpeI act15 rc fw | ACTAGTGATTTTTATTTTTTAATTTAATTTATTTG | Cloning act15 (reverse, complement) |
| DM_205_act15 rc HindIII rev | AAGCTTTAAAAAAAATTTTTATTTATTTTTATTTATTTTG | Cloning act15 (reverse, complement |
