# Supplementary material for: A Simple Retroelement Based Knock-Down System in Dictyostelium: Further Insights into RNA Interference Mechanisms
Source: PLoS One. 2015 Jun 25;10(6):e0131271. doi: 10.1371/journal.pone.0131271 (PMC4482531; doi:10.1371/journal.pone.0131271)
Supplement: S2 Table — (DOCX) [file pone.0131271.s004.docx]

**Table S2:** Trigger sequences and gene names.

| **Gene name*** | **Position of trigger sequence**** | **Sequence length** |
| --- | --- | --- |
| corA DDB_G0267382 | +13; +663 | 650 bp |
| sevA DDB_G0289327 | +897; +1309 | 412 bp |
| abpA DDB_G0268632 | +454; +1168 | 714 bp |
| culD DDB_G0292794 | +1880; +2400 | 520 bp |
| casK DDB_G0276885 | +668; +1180 | 512 bp |
| qtrt1 DDB_G0291802 | +306; +927 | 621 bp |
| mhcA DDB_G0286355 | +1788; +2275 | 487 bp |

* Gene names and accession numbers are given quoting http://dictybase.org/ [[17](#_ENREF_17)].

** Start and end nucleotide relative to the ATG start codon of the gene.
